# Screening of wild roe deer populations in Sweden 2016–2022 for SARS-CoV-2

**DOI:** 10.1016/j.onehlt.2026.101370

**Published:** 2026-02-19

**Authors:** Andrea Berglund, Gustav Averhed, Aleksija Neimanis, Ellinor Spörndly-Nees

**Affiliations:** a Swedish University of Agricultural Sciences, Department of Animal Biosciences, Uppsala, Sweden; b Swedish Board of Agriculture, Jönköping, Sweden; c Swedish Veterinary Agency, Department of Pathology and Wildlife Diseases, Uppsala, Sweden

**Keywords:** Reverse zoonosis, Roe deer (*Capreolus capreolus*), SARS-CoV-2, Wildlife health surveillance, Zoonosis, Cross-species transmission risk

## Abstract

Severe acute respiratory syndrome coronavirus 2 (SARS-CoV-2) has caused a global pandemic with significant human impact and zoonotic concerns. In North America, white-tailed deer (*Odocoileus virginianus*) show high prevalence of infection and viral mutations, raising concerns about novel variants and reverse zoonosis. The World Organisation for Animal Health (WOAH) stated that cervids could become silent reservoirs, urging global surveillance. In Europe, studies have not found significant spill-over into wild deer populations, with negative results in red, roe, fallow, and other deer species across Poland, Germany, Austria, and the UK. However, seropositivity was recently detected in urban fallow deer in Ireland. To assess SARS-CoV-2 presence in Swedish roe deer (*Capreolus capreolus*), retropharyngeal lymph nodes from 110 individuals (2016–2022) were tested for viral nucleic acid. All samples were negative, suggesting limited or no spill-over in Sweden. These findings align with broader European data but contrast with North American trends, highlighting the importance of continued wildlife monitoring.

## Highlights

• First SARS-CoV-2 screening in Swedish roe deer (*Capreolus capreolus*).
• 110 retropharyngeal lymph node samples tested from 2016 to 2022.
• All samples negative for viral RNA, indicating no spill-over detected.
• Results align with European trend, contrasting North American findings.
• Emphasizes need for ongoing wildlife surveillance in Sweden.

## 1 Introduction

Severe acute respiratory syndrome coronavirus 2 (SARS-CoV-2) caused a pandemic with almost 800 million human cases and over 7 million deaths globally [1]. The pandemic likely began with a direct zoonotic viral spillover from an animal host species to humans or via infection of an intermediate animal host followed by spillover to humans. [2].

Since it was first detected, the virus has infected multiple wild [3] and domestic animals. Spillover of SARS-CoV-2 from humans to white-tailed deer (*Odocoileus virginianus*), was demonstrated in North America [4] where high prevalence of the virus has been recorded in some populations of white-tailed deer [5]. Initially it appeared that the virus circulating in deer had not significantly mutated [5], but it was later determined that lineages of the virus circulating in white tailed deer mutate with high frequency [6] which increases the risk of new variants emerging. It has been shown that white-tailed deer can act as a reservoir for SARS-CoV-2 variants of concern (VOC) that no longer circulate among humans. This, coupled with the risk of mutations and reinfection of humans [6], makes new wildlife reservoirs a clear threat to human health. Host adaptation and evidence of unsustained deer to human transmission of a divergent SARS-CoV-2 variant have been reported from Canada [6].

In experiments where white-tailed deer were inoculated with SARS-CoV-2, the virus was found in several organs, including pharyngeal lymph nodes [7]. Roe deer (*Capreolus capreolus*) are related to white-tailed deer [8] and have angiotensin-converting enzyme 2 (ACE 2) receptors for SARS-CoV-2 in their respiratory tract [9].

At the end of 2021, the World Organisation for Animal Health (WOAH) made a statement [5] that white-tailed deer may become a silent reservoir of SARS-CoV-2, so monitoring and research is needed. Further the WOAH statement encouraged national veterinary services and wildlife authorities to monitor and test cervid populations in all regions to further understand the spread of infection within the white-tailed deer population and among other wildlife species, and report this to WOAH [5]. In response to growing global concerns about SARS-CoV-2 in wildlife, a joint statement issued in 2022 by WHO, FAO, and WOAH further highlighted the need to monitor SARS-CoV-2 in wildlife species [10].

To date, there is limited information on the susceptibility of European deer species to SARS-CoV-2 infections.

To better understand the potential deer exposure to SARS-CoV-2 in Sweden, we analysed retropharyngeal lymph nodes (RPLN) from 110 roe deer submitted to the Swedish Veterinary Agency (SVA) for examination. The material in this study came from SVA's biobank and was collected before and after the first human case of SARS-CoV-2 in Sweden.

## 2 Materials and methods

### 2.1 Sample collection and study area

**Fig. 1:**
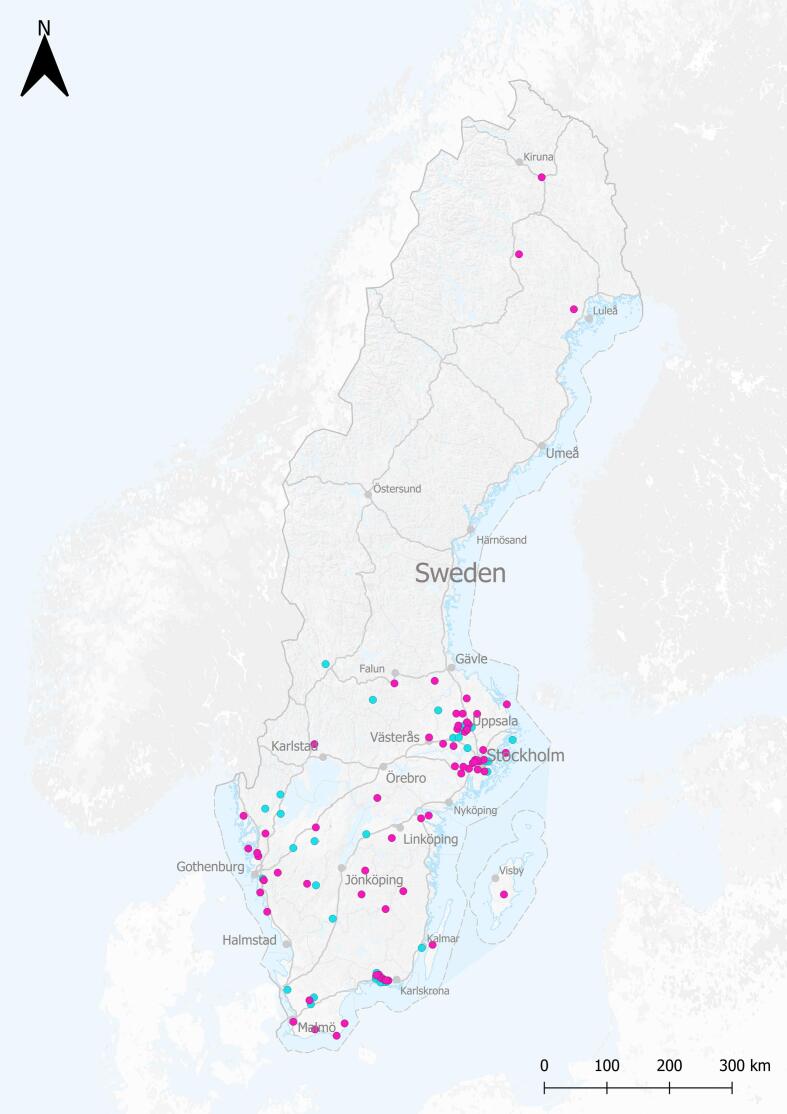
Map of Sweden showing sample location of roe deer (*Capreolus capreolus*). Blue points indicate animals sampled before the first human case of SARS-CoV-2 in Sweden (2 June 2016–11 December 2019). Pink points indicate animals sampled after the first human cases of SARS-CoV-2 in Sweden (24 January 2020–9 March 2022). The black bar indicates 300 km distance. (Map produced by the Swedish Board of Agriculture/ geodata unit: 2025-11-05). (For interpretation of the references to colour in this figure legend, the reader is referred to the web version of this article.)

Between 2 June 2016 and 9 March 2022 free-ranging roe deer were sampled as part of the EU surveillance program for Chronic Wasting Disease (CWD) and national wildlife disease surveillance at SVA. Although samples were collected from roe deer across the country, they mainly originated from southeast Sweden with clusters around Uppsala, Stockholm and Ronneby. Only three individuals came from the northern parts of Sweden (Fig. 1). This coincides with roe deer range and density in Sweden based on reported sightings [11].

The sampled roe deer originated from two sources: Animals found dead or euthanised because of illness (fallen wildlife) (*n* = 98) and those killed in road traffic accidents (roadkill) (*n* = 12). Material submitted to SVA consisted of the entire carcass, only the head or only a sample of the brain stem and cranial lymph nodes. The latter types of material were more common if the distance to SVA was far. Roadkill deer were submitted by search-and-recovery hunters. These animals are considered healthy prior to the incident. All animals underwent CWD testing.

In total, 110 roe deer were tested for SARS-CoV-2. Sexes were equally represented; females (*n* = 55), males (*n* = 52) and unspecified (*n* = 3). The samples were collected before (2 June 2016–11 December 2019; *n* = 38) and after (24 January 2020–9 March 2022; *n* = 72) the first human case of SARS-CoV-2 in Sweden. Sweden's first confirmed COVID-19 case returned to Sweden from Wuhan, China on January 24, 2020.

The majority of sampled roe deer (*n* = 105) were older than two years, four roe deer were less than two years old and age was unknown for one animal. No evidence of CWD was found in any animals. Following examination and/or sampling for CWD, tissues were archived in SVA's biobank at −20 °C until thawed for sampling and immediate processing for molecular analysis.

### 2.2 Sample collection

A retropharyngeal lymph node from each animal was thawed, sliced with a sterile scalpel blade and the cut surface was swabbed, ensuring no cross-contamination between animals. The presence of SARS-CoV-2 nucleic acid was assessed by a real-time PCR assay. Swabs were analysed in the Department of Microbiology, SVA, Uppsala, Sweden for SARS-CoV-2 PCR. After RNA extraction, real-time, reverse transcriptase PCR (RT-qPCR) targeting the RdRP gene was performed according to [12] with minor modifications.

## 3 Results

No SARS-CoV-2 RNA was detected in any of the 110 sampled roe deer using RT-PCR. The estimated prevalence for SARS-CoV-2 in the investigated roe deer population was 0%. Lower and upper 95% confidence limits were 0% - 2.7%. Therefore, prevalence was determined to be less than 2.7% with 95% probability, given perfect diagnostic tests.

## 4 Discussion

This study found no evidence of SARS-CoV-2 RNA in any roe deer examined. Results indicate that spillover infection of SARS-CoV-2 to the roe deer population in Sweden was absent to limited during the study period. Notably SARS-CoV-2 had only been endemic in Sweden for approximately two years during the latter part of the sampling and data collection period. This relatively short duration may have limited our ability to fully evaluate potential viral spillover into cervid populations. We cannot completely rule out that roe deer in Sweden were infected or carried the virus, as the number of roe deer examined was relatively small. However, the results do not support widespread occurrence of the virus in roe deer in Sweden.

The first Eurasian case of SARS-CoV-2 seropositivity in wild ruminants was found in a free-ranging urban population of wild fallow deer (*Dama dama)* in Dublin, Ireland [13]. Research studies from other European countries have not shown evidence of exposure to SARS-CoV-2 in European deer and wild ruminant species. A 2022 study from Northeastern Poland found no evidence that wild red deer *(Cervus elaphus)* play a role as vector or reservoir of SARS-CoV-2 [14]. Similarly, a German study did not find any evidence of SARS- CoV-2 in wild ruminant species: fallow deer*,* mouflon (*Ovis gmelini*), red deer, roe deer and wisent (*Bison bonasus*) [15]. A 2022 study conducted in the United Kingdom found no evidence of SARS-CoV-2 in UK deer: Chinese water deer *(Hydropotes inemis),* fallow deer, muntjac *(Muntiacus reevesi),* red deer, red/sika hybrid, roe deer, or sika (*Cervus nippon*) during the study period [16]. Another 2022 study conducted in roe deer, red deer and fallow deer in Germany and Austria found no evidence of SARS-CoV-2 exposure [17].

Apart from the positive findings in fallow deer in Ireland, our study results are in line with other European countries' reports in different wild ruminant species [14], [15], [16], [17] but differ from those conducted in North America [3], [4], [5], [18], [19], [20] where a high prevalence of SARS-CoV-2 has been reported in white-tailed deer.

Different factors may have influenced the results of this study. Interactions with wildlife, and in particular with deer populations, may differ between countries. Also, close contact with humans may differ between species and settings (e.g. in nature versus wildlife parks). To date, there is no or limited information on the interaction between humans and wild roe deer in Sweden. However, roe deer often inhabit peri-urban and urban environments.

Additionally, different methods were used in the present and previous studies, which may impact results. In the present study, retropharyngeal lymph nodes were used for direct detection of SARS-CoV-2 nucleic acid. The period when SARS-CoV-2 virus nucleic acid is present and detectable during an infection may be short. Methods in previous studies included nasal swabs for detection of viral RNA and serology for antibody detection which may pick up cases not detected in this study. Variations in the sensitivity and specificity of diagnostic methods also can influence observed prevalence and should be considered when comparing different studies. To improve understanding of both current and past infections, future studies on SARS-CoV-2 in wildlife in Sweden should include serological testing for antibodies alongside PCR-based detection of viral RNA. The material used in this study originates from targeted surveillance for CWD, and although designed to maximize geographic representation, samples were not collected from all parts of the roe deer population range. The collection of material to be tested was based on voluntary collection, which may bias geographical distribution of samples. In addition to medial retropharyngeal lymph nodes, Palmer et al. [7] found high viral loads in nasal turbinates and palatine tonsils. These tissues were not available in this study, and it is possible that viral presence may have been missed. In future studies, additional tissues could be included.

Other factors, such as time from sampling to analysis, suboptimal transportation and suboptimal storage prior to testing, may affect success of virus isolation in this study. RNA degradation can occur during storage at -20 °C which can lead to false negative results and preclude detection of SARS-CoV-2 infected roe deer. However, the target sequence of the qPCR was very short, minimizing this risk [21]. Other studies using the same storage conditions detected positive cases in deer [4].

Although our study did not detect SARS-CoV-2 in Swedish deer, the global evidence of infection and emerging variants in wild animal populations underscores the critical need for ongoing surveillance of wildlife. Continued monitoring, in line with WHO, FAO, and WOAH recommendations [10], is essential to detect potential new reservoirs and reduce the risk of reverse zoonosis.

## CRediT authorship contribution statement

**Andrea Berglund:** Writing – original draft, Methodology, Investigation, Formal analysis. **Gustav Averhed:** Writing – review & editing, Methodology, Funding acquisition, Data curation. **Aleksija Neimanis:** Writing – review & editing, Supervision, Funding acquisition, Conceptualization. **Ellinor Spörndly-Nees:** Writing – review & editing, Supervision, Methodology, Investigation, Funding acquisition, Formal analysis, Data curation, Conceptualization.

## Ethical statement

No ethical approval was required as tissues/ lymph nodes were derived from fallen wildlife and road kills.

## Author statement

During the preparation of this work, the authors used ChatGPT and Copilot to generate an initial framework for the abstract and, in a few sections, to enhance readability and language. All AI-assisted content was carefully reviewed and edited by the authors, who take full responsibility for the final version.

## Funding

We thank 10.13039/501100004357The Swedish Environmental Protection Agency for funding the study. Grant Number: NV-02625-23.

## Declaration of competing interest

None.

## Data Availability

Data will be made available on request.
